# Prognostic value of the 7-year protocol biopsy of adult kidney allografts: impact of mesangiosclerosis and proteinuria

**DOI:** 10.1080/0886022X.2023.2197499

**Published:** 2023-04-11

**Authors:** Yoshihiro Itabashi, Hideyo Oguchi, Tetuo Mikami, Noriyuki Kounoue, Taichi Arai, Kazunobu Shinoda, Masaki Muramatsu, Seiichiro Shishido, Ken Sakai

**Affiliations:** a Department of Nephrology, Toho University Faculty of Medicine; b Department of Pathology, Toho University Faculty of Medicine; c Department of Urology, St. Marianna University School of Medicine

**Keywords:** Kidney transplantation, 7-year protocol biopsy, Banff score, allograft survival

## Abstract

**Aim:**

The aim of the present study was to clarify the relationship between the Banff score of the 7-year protocol biopsy and the allograft outcome.

**Methods:**

One-hundred-and-eighty-four patients received kidney transplantation from 2002 to 2008. We excluded patients aged <20 years at transplantation (*n* = 24), those who did not undergo a 7-year protocol biopsy (*n* = 66), and those who underwent for-cause biopsy (*n* = 5). Consequently, 89 patients who underwent a 7-year protocol biopsy were enrolled. We analyzed the relationship between the clinicopathological findings 7 years after transplantation and the estimated glomerular filtration rate (eGFR) change per year and allograft survival. Histological evaluation was performed using the Banff 2015 classification.

**Results:**

Among the clinicopathological findings, each Banff mesangial matrix increase (mm) score ≥1 and proteinuria ≥1+ was independently associated with the eGFR decline per year during a median follow-up of 73 months. Furthermore, in the model of the clinicopathological findings including the presence of mm with proteinuria, mm ≥1 alone and mm ≥1 with proteinuria were each independently associated with the eGFR decline. The graft survival was significantly worse for those with mm ≥1 with proteinuria than those with mm ≥1 without proteinuria.

**Conclusion:**

Among the 7-year protocol biopsy findings, the presence of mm alone and mm with proteinuria were each significant predictors of eGFR decline. The presence of both proteinuria and mm had a negative impact on graft survival. These results underscore the significance of the Banff mm score and proteinuria at the time of the 7-year protocol biopsy to predict the allograft outcome.

## Introduction

The Organ Procurement and Transplantation Network (OPTN)/Scientific Registry of Transplant Recipients (SRTR) 2019 annual report stated that maintaining long-term kidney graft survival is still challenging [1]. A previous report suggested that the long-term protocol biopsy has potential significance in detecting non-immunological factors that may lead to the deterioration of allograft function [2], although there has been little evidence supporting the usefulness of the long-term protocol biopsy in detecting chronic allograft rejection. Nankivell et al. [3] demonstrated that chronic allograft nephropathy progressively develops by 10 years post-transplantation in the natural course after kidney transplantation, with about 60% of cases severely affected in 10 years regardless of preceding rejection episodes. Furthermore, another study of 145 patients who underwent protocol biopsies at both 5 and 10 years after kidney transplantation showed that 54% of patients at 5 years and 82% of patients at 10 years had non-immune pathological injuries including arteriolar hyalinosis, mesangial sclerosis, and glomerulosclerosis [4]. As these changes presumably lead to allograft dysfunction, it may be necessary to perform protocol biopsies earlier than 10 years after transplantation.

To the best of our knowledge, only a few studies have analyzed the relationship between the long-term protocol biopsy findings and the allograft outcome. In a study investigating the relationship between the histology of the 10-year protocol biopsy and allograft outcome, multivariate analysis showed that muscular arteriolopathy at the 10-year biopsy is not a significant predictor of allograft loss [5]. Recently, the Banff score has been improved and revised to evaluate acute or chronic lesions of the glomeruli, peritubular capillaries, tubules, interstitium, and arterioles in detail [6]. However, the significance of the Banff score itself, not the Banff classification, of the long-term protocol biopsy in predicting allograft outcomes has not yet been clarified in clinical practice.

In the present study, we focused on the 7-year protocol biopsy to investigate histological changes that occur prior to the 10-year protocol biopsy. The purpose of the present study was to evaluate the significance of the Banff scores of the 7-year protocol biopsy in predicting the allograft outcome.

## Materials and methods

### Population and study protocols

**Figure 1.**
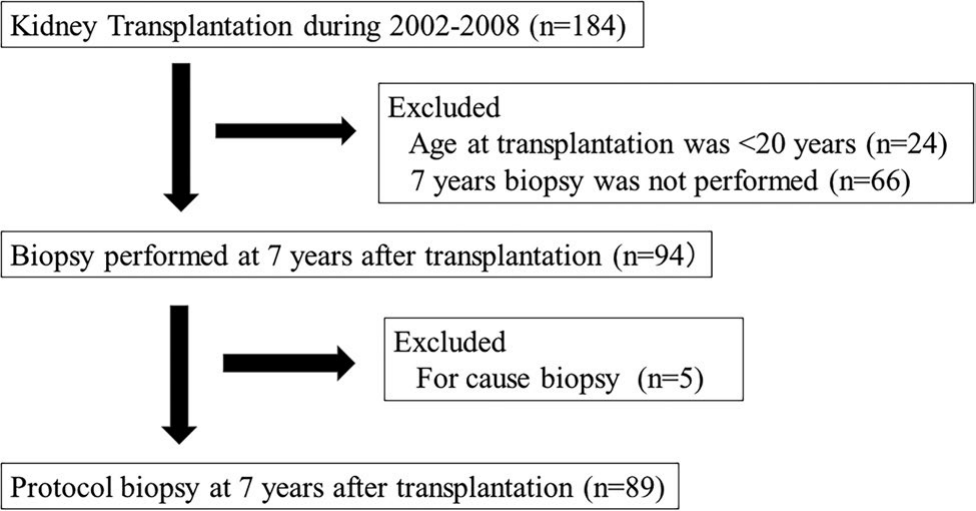
Flowchart of patients undergoing the 7-year protocol biopsy.

The present study was a retrospective analysis of a cohort treated in a single center. We performed the 7-year protocol biopsy within 6–8 years after kidney transplantation. The process of patient selection is demonstrated in Figure 1. One-hundred-and-eighty-four patients received kidney transplantation from 2002 to 2008 in the Toho University Omori Medical Center. We excluded patients aged <20 years at transplantation (*n* = 24), those who did not undergo a 7-year biopsy (*n* = 66), and those who underwent a for-cause biopsy during the same period (*n* = 5). The final study cohort comprised 89 patients who underwent a protocol biopsy at 7 years after kidney transplantation. We used basiliximab for the induction therapy, and the maintenance oral immunosuppressants included calcineurin inhibitors (CNI) (tacrolimus or cyclosporine), methylprednisolone, and antimetabolites (mycophenolate mofetil or mizoribine). We routinely performed protocol biopsies at 1 h, 3 months, 1 year, 5 years, 7 years, and 10 years after kidney transplantation. The biopsy samples were collected using a 16 G biopsy needle.

### Clinical and pathological data

We reviewed the medical records to collect clinical characteristics including recipient and donor ages, sex, cold ischemic time, an original disease that caused end-stage renal failure, and the prevalence of a cadaveric donor, HLA mismatch, second transplantation, and ABO-incompatible transplantation. The information about the administration of CNI, other immunosuppressive treatments, use of a renin-angiotensin system inhibitor, use of diuretics, steroid pulse therapy, cytomegalovirus viremia, estimated glomerular filtration rate (eGFR), proteinuria, and comorbid diabetes and hypertension were obtained at the time of the 7-year protocol biopsy. The eGFR was calculated using the Japanese eGFR formula [7]. We assessed the relationship between the clinicopathological characteristics and eGFR change per year. We calculated the eGFR change per year as the difference between the eGFR at the time of the biopsy and the most recent eGFR obtained during follow-up until July 2019 divided by the time. In the case of the start of renal replacement therapy, we adopted the eGFR at the time of the induction of renal replacement therapy. Proteinuria was assessed qualitatively. The effects of malignancy, non-adherence, and cardiovascular disease which was defined as the presence of stroke, angina, or myocardial infarction were also analyzed.

The histological specimens were evaluated using light microscopy in accordance with the Banff 2015 classification [6]. The Banff double counter (cg) score was diagnosed using light microscopy, although the recent Banff classification states that the evaluation should be done using electron microscopy [8]. The Banff score was evaluated in an unblinded manner by one nephrologist (HO) and one pathologist (TM). We defined acute/chronic active pathological antibody-mediated rejection (ABMR) as a light microscopic specimen that histologically met the Banff criteria regardless of the presence of donor-specific anti-HLA antibody (DSA), based on our previous report [9]. We defined ‘IgA deposition’ as IgA positivity not accompanied by urinary abnormalities, and defined ‘IgA nephropathy’ as IgA positivity accompanied by urinary abnormalities, based on the previous report [10]. Arteriolar hyalinosis was diagnosed based on arteriolar hyalinosis (ah)≥2 or hyaline arteriolar thickening (aah)≥2 without histological evidence of ABMR, IgA deposition, IgA nephropathy, or diabetic nephropathy. Diabetic nephropathy was defined as the presence of diabetes and pathological features, as previously reported [11].

### Statistical analysis

We performed statistical analyses using SPSS version 23 (IBM, JAPAN), with *p* < 0.05 defined as statistically significant. All data were expressed as the median (interquartile range (IQR)) or number (%). We evaluated the association between the eGFR change per year and clinicopathological variables using simple linear regression analysis. We also performed multivariate linear regression analysis to identify independently significant variables using two models. In the pathological model (model 1), we performed multivariate linear regression analysis using covariates that were significant pathological factors in simple linear regression analysis. In the clinicopathological mixed model (model 2), we performed multivariate linear regression analysis using the same covariates as in model 1 plus the covariates that were significant clinical factors in simple linear regression analysis. Death-censored graft survival was analyzed by the log-rank test.

## Results

### Baseline clinical characteristics and histological findings of 7-year protocol biopsies

Table 1 shows the baseline clinical and histological characteristics of the 7-year protocol biopsies (*n* = 89). The median (IQR) recipient age and donor age were 40 (31, 53) years and 57 (52, 64.5) years, respectively. The numbers of male/female recipients and donors were 49/40 and 42/47, respectively. The prevalences of a cadaveric donor, second transplantation, and ABO-incompatible transplantation were 4.5%, 6.7%, and 19.1%, respectively. The median (IQR) eGFR at the time of the biopsy was 44.5 (33.1, 53.3) mL/min per 1.73 m^2^, and the prevalence of proteinuria ≥ 1+ at the time of the biopsy was 14.6%. The prevalences of diabetes and hypertension at the time of the biopsy were 23.6% and 83.1%, respectively. DSA was measured in 16 of 89 cases (18.0%); nine of the 16 cases (56.3%) were defined as positive (highest mean fluorescence intensity was ≥1000) in accordance with our institution protocol [9] (DSA data not shown in Tables). The C7HRP was measured in nine of 89 patients, two of whom had positive results (both two cases were 1/50000). The background of patients were compared between included patients and excluded patients who did not have a 7-year protocol biopsy. The prevalence of second or more transplantation was significantly higher, and the prevalence of diabetic nephropathy as original disease was significantly lower in the included patients group (Supplemental Table 1).

**Table 1.** Baseline clinical characteristics of patients who underwent 7-year protocol biopsies.

	All cases (*n* = 89)
Clinical findings	
Recipient age, years	40 (31, 53)
Recipient sex, (male/female), *n*	49/40
Donor age, years	57 (52, 64.5)
Donor sex, (male/female), *n*	42/47
Cadaveric donor, *n* (%)	4 (4.5)
HLA mismatches, *n*	3 (2, 3)
Second transplantation, *n* (%)	6 (6.7)
ABO-incompatible, *n* (%)	17 (19.1)
Cold ischemic time (minute)	55 (44, 75)
Original kidney disease	
IgA nephropathy, *n* (%)	15 (16.9)
Diabetic nephropathy, *n* (%)	5 (5.6)
Others, *n* (%)	46 (51.7)
Unknown, *n* (%)	23 (25.8)
TAC/CYA, *n*	41/48
MMF/MZ/AZ/EVR, *n*	74/8/2/2
Use of RASi, *n* (%)	70 (78.7)
Use of diuretics, *n* (%)	4 (4.5)
Steroid pulse therapy, *n* (%)	1 (1.1)
eGFR (ml/min/1.73m^2^)	44.5 (33.1, 53.3)
Urine protein, *n*	(-): 59, (±): 17, (1+): 8, (2+): 4, (3+): 1
Diabetes, *n* (%)	21 (23.6)
Hypertension, *n* (%)	74 (83.1)
CVD, *n* (%)	3 (3.4)
Malignancy, *n* (%)	2 (2.2)
Non-adherence, *n* (%)	4 (4.5)
Pathological findings	
Glomerular sclerosis rate (%)	26.9 (12.3, 42.4)
Acute TCMR, *n* (%)	0 (0)
Acute ABMR, *n* (%)	8 (9.0)
Chronic active ABMR, *n* (%)	10 (11.2)
C4d score (*n* = 69), n	score0: 57, score1: 7, score2: 2, score3: 3
Prior rejection, *n* (%)	6 (6.7)
BKVN, *n* (%)	0 (0)

Data are expressed as median (IQR) or n (%). The data about the CNI and other immunosuppressive agents, eGFR, proteinuria, diabetes, hypertension, and pathological findings were at the time of the 7-year protocol biopsy. TAC: tacrolimus; CYA: cyclosporine; MMF: mycophenolate mofetil; MZ: mizoribine; AZ: azathioprine; EVR: everolimus; RASi: renin-angiotensin-system inhibitor; BKVN: BK virus nephropathy; CMV: cytomegalovirus; CVD: cardiovascular disease; eGFR: estimated glomerular filtration rate; TCMR: T-cell-mediated rejection; ABMR: antibody-mediated rejection.

**Figure 2.**
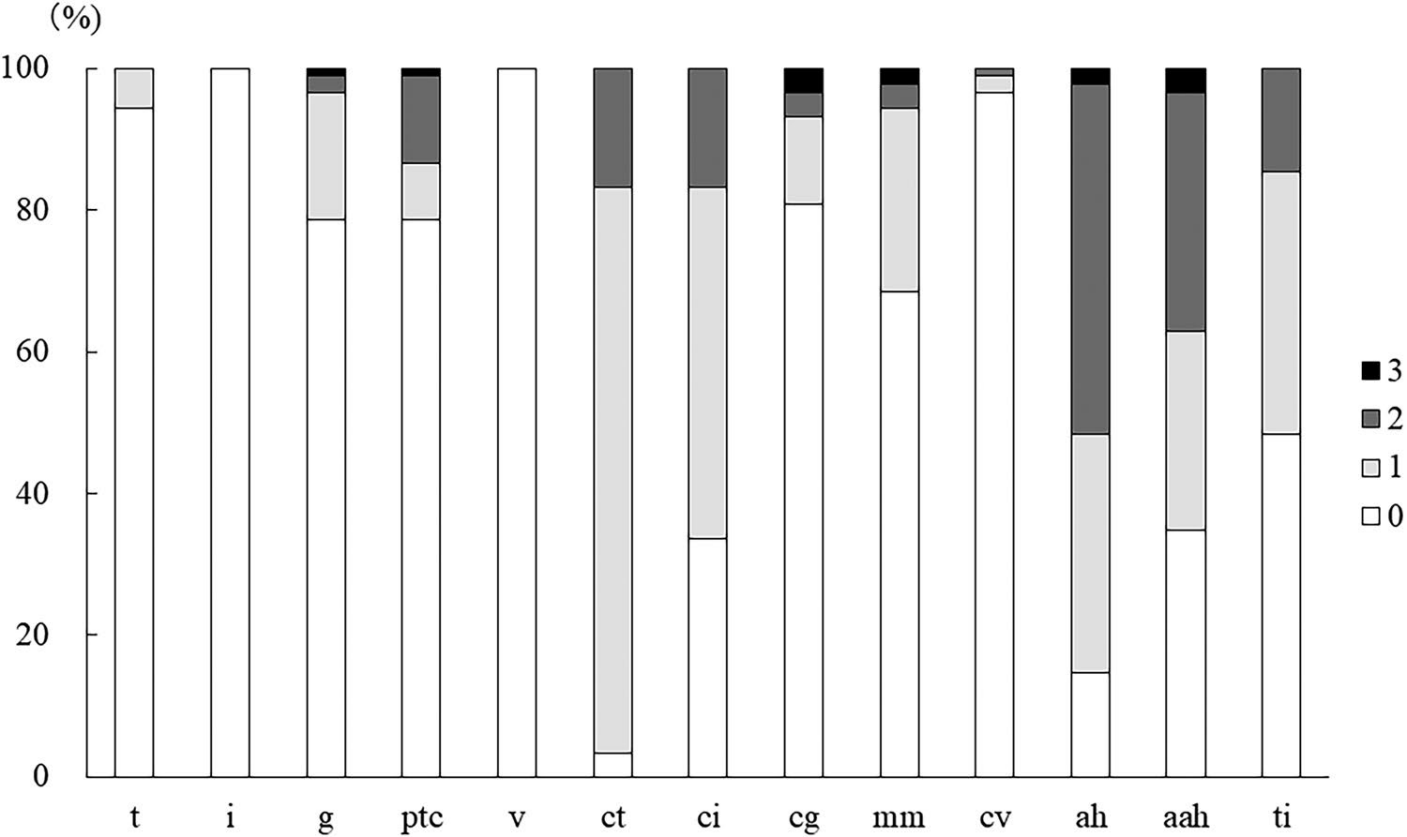
Prevalence of positive Banff scores for the 7-year protocol biopsy.

The distribution of Banff scores for the 7-year protocol biopsies is shown in Figure 2. The prevalence of positive score for tubulitis (*t* ≥ 1) was 6%. The prevalences of positive scores for glomerulitis (*g* ≥ 1) and peritubular capillaritis (ptc ≥ 1) were both 21%. The prevalences of chronic scores were 97% for tubular atrophy (ct ≥ 1), 66% for interstitial fibrosis (ci ≥ 1), 18% for cg ≥ 1, 31% for mesangial matrix expansion (mm ≥ 1), 3% for vascular fibrous intimal thickening (cv ≥ 1), 85% for ah ≥ 1, 65% for aah ≥ 1 and 52% for total inflammation (ti ≥ 1).

### Uni- and multivariate linear regression analyses for the association between eGFR change per year and clinicopathological findings

The median (IQR) follow-up of the eGFR change per year was 73 (54.5, 97.0) months. Table 2 shows the univariate analysis results for the association between the eGFR change per year and the clinicopathological findings. Among the pathological findings, Banff t, g, ptc, ct, ci, cg, mm, and ah scores of ≥1 were significantly associated with the annual eGFR decline. Among the clinical findings, the eGFR and proteinuria ≥1+ at the 7-year biopsy were each significantly associated with the annual eGFR decline.

**Table 2.** Linear univariate regression analysis results for the association between eGFR change per year and each parameter.

	Univariate		
	β	Coefficient	*p* value
Pathological findings			
glomerular sclerosis rate	0.053	0.007	0.629
t score ≥1	−0.214	−2.375	0.047
g score ≥1	−0.380	−2.382	<0.001
ptc score ≥1	−0.364	−2.281	0.001
ct score ≥2	−0.375	−2.565	<0.001
ci score ≥2	−0.375	−2.565	<0.001
cg score ≥1	−0.412	−2.692	<0.001
mm score ≥1	−0.508	−2.815	<0.001
cv score ≥1	−0.122	−1.730	0.260
ah score ≥2	−0.278	−1.436	0.009
aah score ≥2	−0.176	−0.946	0.102
ti score ≥2	−0.108	−0.784	0.319
C4d score ≥2	−0.051	−0.537	0.684
Prior rejection	−0.426	−4.345	<0.001
Clinical findings			
Recipient age	0.071	0.015	0.513
Recipient sex	−0.182	−0.945	0.092
Donor age	−0.162	−0.040	0.133
Donor sex	0.101	0.523	0.352
Cadaveric donor	−0.030	−0.372	0.782
HLA mismatches	−0.173	−0.324	0.110
Second transplantation	0.085	0.868	0.434
ABO-incompatible	−0.039	−0.262	0.719
Tacrolimus/cyclosporine	−0.041	−0.216	0.703
eGFR	0.311	0.047	0.003
Proteinuria ≥1+	−0.472	−3.428	<0.001
Diabetes	0.024	0.147	0.823
Hypertension	−0.124	−0.847	0.254
Use of RASi	−0.077	−0.482	0.479
Use of diuretics	−0.042	−0.514	0.702
Steroid pulse therapy	0.090	2.185	0.407
CVD	0.069	0.984	0.523
Malignancy	−0.053	−0.922	0.623
Non-adherence	–0.053	–0.653	0.627

The data of pathological findings, CNI, eGFR, proteinuria, diabetes, and hypertension, use of RASi, use of diuretics, and steroid pulse therapy were collected at the time of the 7-year protocol biopsy. eGFR: estimated glomerular filtration rate; RAS renin-angiotensin system inhibitors.

Table 3 demonstrates the multivariate analysis results of covariates associated with the annual eGFR decline. In the multivariate analysis, Banff ct score ≥1 was excluded because of multicollinearity. In model 1, the Banff mm score ≥1 was significantly associated with the annual eGFR decline (β= −0.292, coefficient= −1.620, *p* = 0.007). In model 2, mm ≥1 and proteinuria ≥1+ were each significantly associated with the annual eGFR decline (mm ≥1: β=-0.273, coefficient= −1.510, *p* = 0.008, proteinuria ≥1+: β= −0.271, coefficient= −1.970, *p* = 0.004).

**Table 3.** Linear multivariate regression analysis results for the association between eGFR change per year and each parameter.

	Multivariate			
	β	Coefficient	p value	VIF
model 1				
t score ≥1	−0.145	−1.606	0.135	1.269
g score ≥1	0.003	0.021	0.979	2.099
ptc score ≥1	−0.208	−1.305	0.057	1.608
ci score ≥2	−0.151	−1.032	0.150	1.487
cg score ≥1	−0.107	−0.698	0.339	1.718
mm score ≥1	−0.292	−1.620	0.007	1.528
ah score ≥2	−0.077	−0.396	0.411	1.190
Prior rejection	−0.145	−1.481	0.149	1.374
model 2				
t score ≥1	−0.143	−1.592	0.118	1.279
g score ≥1	−0.038	−0.240	0.747	2.177
ptc score ≥1	−0.166	−1.037	0.112	1.647
ci score ≥2	−0.013	−0.088	0.905	1.798
cg score ≥1	−0.071	−0.463	0.504	1.736
mm score ≥1	−0.273	−1.510	0.008	1.543
ah score ≥2	−0.081	−0.418	0.359	1.192
Prior rejection	−0.109	−1.115	0.252	1.391
eGFR	0.116	0.018	0.202	1.273
Proteinuria ≥1	−0.271	−1.970	0.004	1.296
model 3				
t score ≥1	−0.160	−1.774	0.077	1.289
g score ≥1	−0.047	−0.296	0.684	2.180
ptc score ≥1	−0.159	−0.994	0.119	1.649
ci score ≥2	0.018	0.121	0.868	1.831
cg score ≥1	−0.040	−0.261	0.702	1.770
mm score ≥1	−0.213	−1.182	0.038	1.669
ah score ≥2	−0.086	−0.446	0.317	1.193
Prior rejection	−0.078	−0.792	0.410	1.427
eGFR	0.104	0.016	0.246	1.279
Proteinuria ≥1+	−0.116	−0.840	0.319	2.166
mm ≥1 with proteinuria ≥1+	−0.275	−2.617	0.036	2.717

The data of eGFR and proteinuria were at the time of the 7-year protocol biopsy. VIF, valiance inflation factor; eGFR, estimated glomerular filtration rate.

An additional multivariate analysis was performed using model 3, which included the covariates in model 2 plus the mm score ≥1 with proteinuria. In model 3, the Banff mm score ≥1 alone and mm ≥1 with proteinuria were each independently associated with the annual eGFR decline (mm ≥1: β= −0.213, coefficient −1.182, *p* = 0.038; mm ≥1 with proteinuria: β=-0.275, coefficient −2.617, *p* = 0.036). In models 1–3, the variance inflation factor showed no evidence of multicollinearity other than for the ct score.

**Figure 3.**
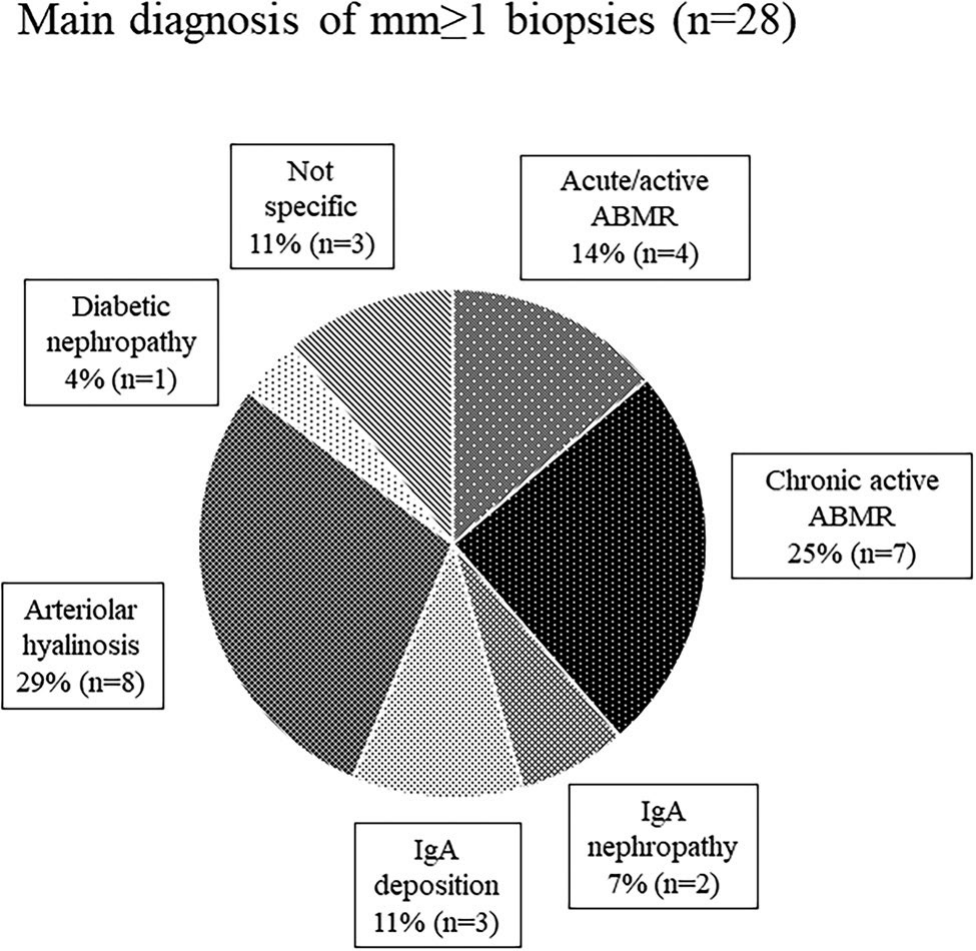
Main diagnoses of mm-positive lesions.

Figure 3 shows the main diagnosis of mm ≥ 1 cases to identify the diseases affecting mm. The prevalence of mm with ABMR was 39% among all mm-positive cases.

### Graft survival

**Figure 4.**
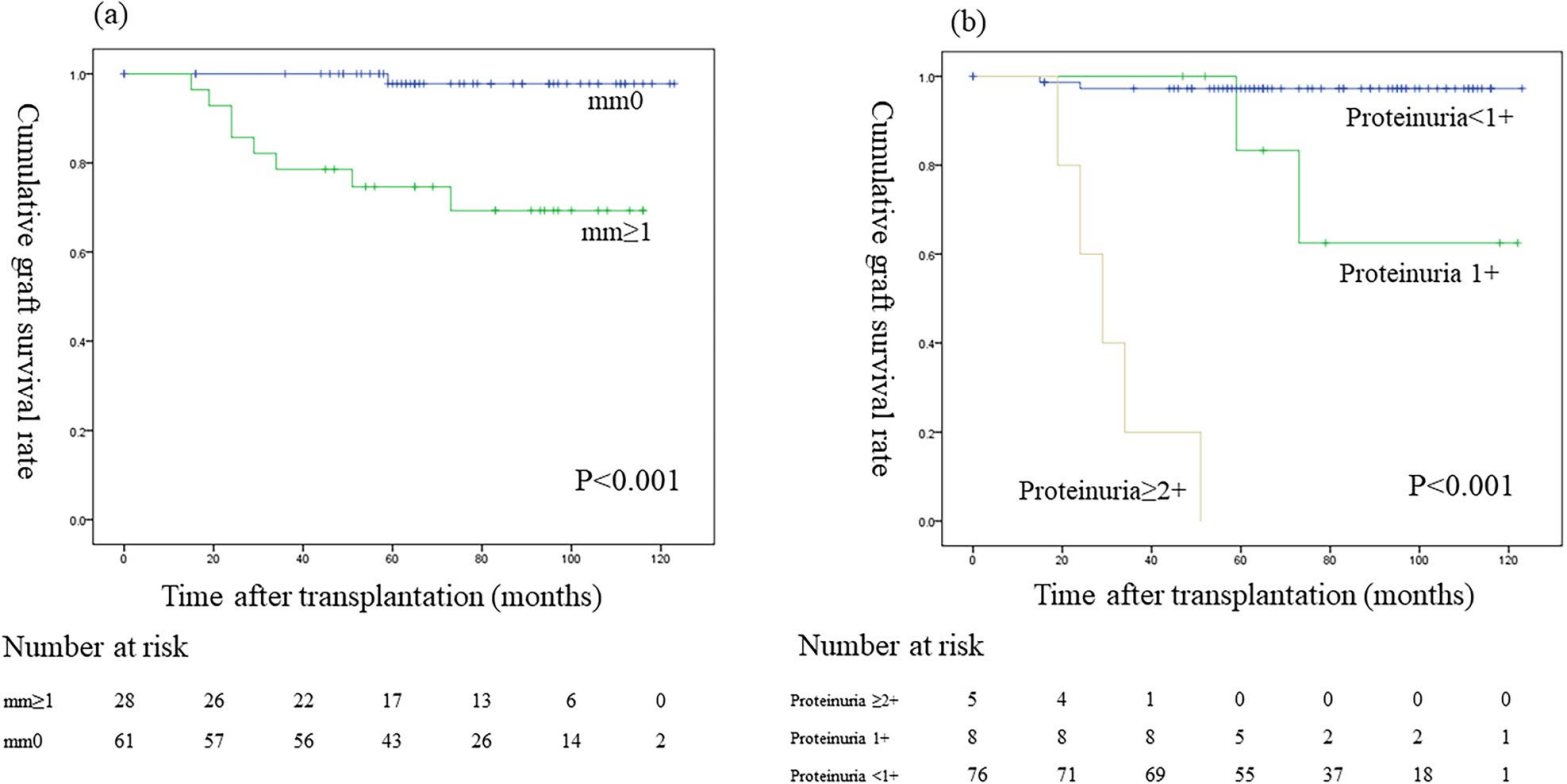
Graft survival between (a) mm < 1 and mm ≥ 1; (b) proteinuria < 1+, proteinuria 1+, and proteinuria ≥ 2+.

**Figure 5.**
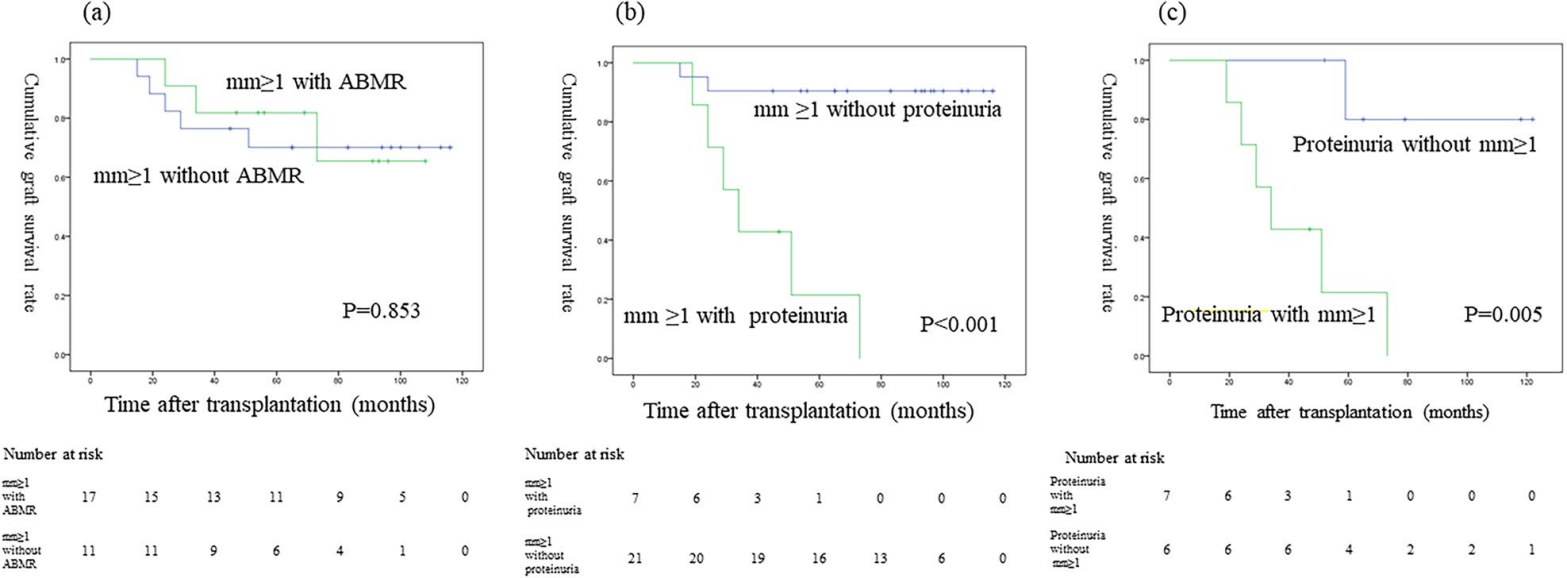
Graft survival between (a) mm ≥ 1 with ABMR and mm ≥ 1 without ABMR; (b) mm ≥ 1 with proteinuria and mm ≥ 1 without proteinuria; (c) proteinuria with mm ≥ 1 and without mm ≥ 1.

Nine of the 89 patients (10.1%) who underwent a 7-year protocol biopsy subsequently lost graft function. Death-censored graft survival curves comparing mm0 vs mm ≥ 1 showed that the graft survival was significantly worse for mm ≥ 1 than mm0 (*p* < 0.001; Figure 4(a)). Death-censored graft survival curves showed that the graft survival significantly differed among patients with ≥2+, 1+, and negative proteinuria results (*p* < 0.001; Figure 4(b)). Death-censored graft survival curves showed that the graft survival did not significantly differ between mm ≥ 1 with and without ABMR (*p* = 0.853; Figure 5(a)). Death-censored graft survival curves showed that the graft survival was significantly worse for mm ≥ 1 with proteinuria versus without proteinuria (*p* < 0.001; Figure 5(b)). Death-censored graft survival curves showed that the graft survival was significantly worse for proteinuria with mm ≥ 1 than without mm ≥ 1 (*p* = 0.005; Figure 5(c)). Comparisons of the graft survival in association with other histological findings are shown in the supplementary text and figures (Figures S1–3).

**Figure 6.**
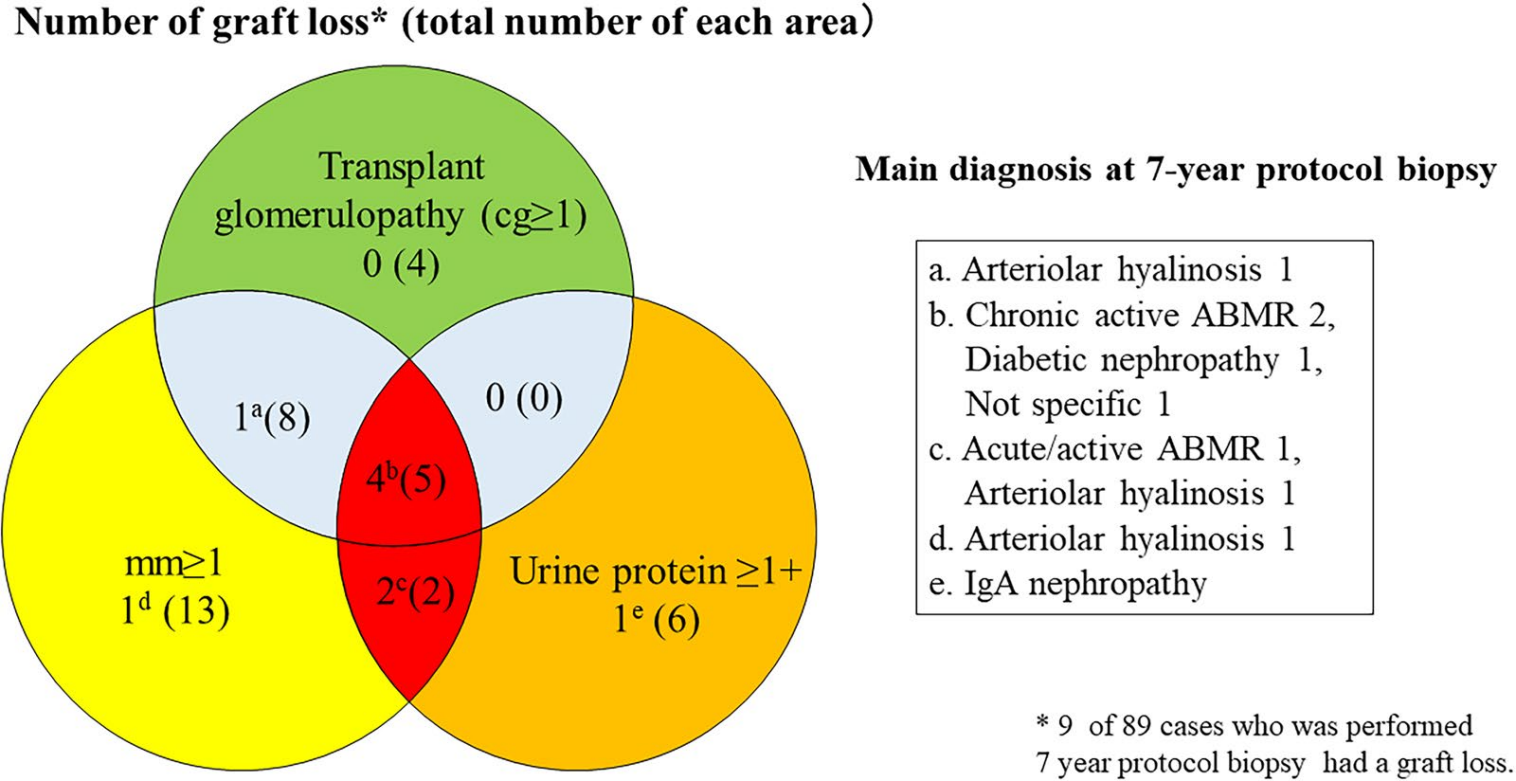
Overlap among graft loss cases with mm ≥ 1, cg ≥ 1, and proteinuria ≥ 1+.

The Banff mm score was evaluated by the degree of mesangial matrix increase, while the Banff cg score was evaluated by glomerular basement membrane duplication [12]. The histological finding of transplant glomerulopathy included glomerular basement membrane duplication following mesangial matrix increase [13]. Figure 6 shows the numbers of patients with graft loss among those with mm ≥ 1, cg ≥ 1, and proteinuria ≥ 1. Six of seven patients with graft loss had an mm-positive lesion with proteinuria.

## Discussion

In the present study of 89 patients, we evaluated the rationale of performing the 7-year protocol biopsy to obtain histological results earlier than the 10-year biopsy. Multivariate analysis found that the Banff mm score ≥1 alone and mm ≥ 1 with proteinuria were each independent predictors of annual eGFR decline during a median follow-up of 73 months. Patients with mm ≥ 1 had significantly worse allograft survival than those with mm0. Furthermore, among patients with mm ≥ 1, proteinuria had a significant negative impact on graft survival. Thus, the present results revealed the significance of the Banff mm score and proteinuria at the time of the 7-year protocol biopsy in predicting the allograft outcome, and highlighted the clinical significance of performing the 7-year protocol biopsy.

It is important to determine whether each Banff score assessing acute or chronic tissue injury findings is a significant predictive factor for the kidney allograft outcome. Our study showed that only the Banff mm score had a negative impact on both the eGFR decline and graft survival. The Banff mm score was graded in accordance with the degree of mesangial matrix increase [12], and a previous report stated that mesangial matrix increase and glomerular basement membrane duplication are observed in transplant glomerulopathy [13]. Moreover, Wavamunno et al. [14] reported that the mesangial matrix is markedly increased from 3 years after transplantation in the course of the development of transplant glomerulopathy. Regarding the etiology of mm, our data showed that 39% of mm-positive lesions had ABMR (Figure 3). Similarly, a recent study stated that transplant glomerulopathy affects approximately half of the patients with chronic active ABMR, and the Banff mm score reflects poor graft survival [15].

As shown in Figure 5(a), there was no significant difference in the allograft survival between ABMR-positive and -negative patients in the mm-positive cohort. This implies that the mm induced by factors other than ABMR may also cause graft loss. In our study, the main diagnosis in 28% of mm-positive cases was arteriolar hyalinosis (Figure 3), and three of eight mm-positive patients with graft loss had ­arteriolar hyalinosis (Figure 6). Arteriolar hyalinosis is induced by both tacrolimus and cyclosporine [16], and the finding of mesangial expansion is also observed in cyclosporine-associated chronic nephrotoxicity [17]. The mm lesion induced by chronic CNI nephrotoxicity might cause poor graft outcomes. Additionally, a previous study revealed that mesangial matrix increase occurs in the 5/6 nephrectomy rat model through the overexpression of genes and proteins in the extracellular matrix and leads to glomerular sclerosis [18]. Therefore, mm in kidney allografts may be a potential predictive factor for glomerulosclerosis.

In our study, the concomitant presence of proteinuria with mm had a strong negative impact on eGFR decline and graft survival (Table 3, Figures. 5(b), 5(c), and 6), indicating the importance of evaluating concomitant proteinuria as well as the presence of mm in the 7-year protocol biopsy. Furthermore, four of five mm-positive patients with transplant glomerulopathy and proteinuria experienced graft loss (Figure 6). A recent study of transplant glomerulopathy showed that the level of proteinuria and the presence of mm ≥ 1 affect the graft survival [15]. Electron microscopy reveals endothelial injury in glomeruli in ABMR leading to transplant glomerulopathy [19], and we also recently reported that the electron microscopic findings of glomerular endothelial injury are detected in patients with low-level proteinuria with ABMR [9]. A histological study using electron microscopy revealed that glomerular endothelial injury is the initial change leading to transplant glomerulopathy with mesangial matrix increase [14]. Taken together, these results suggest that it may be important to focus on the treatment of glomerular endothelial injury in patients with mm-positive lesions.

The strength of our study was that it not only provided evidence regarding the relationship between the Banff score of the 7-year protocol biopsy and the allograft outcome, but also showed the impact of the mm score and concomitant proteinuria. In clinical practice, if the 7-year protocol biopsy reveals an mm-positive lesion in a patient without proteinuria, we suggest initiating clinical care to prevent proteinuria. Furthermore, if the patient has an mm-positive lesion and proteinuria at the time of the 7-year protocol biopsy, it is important to initiate anti-proteinuria treatment. However, the present study has several limitations. First, this was a retrospective single-center analysis with a small sample size. Multicenter studies with larger populations are required to validate the present findings. Second, the Banff scores were not blindly assessed. Third, the testing of DSA was evaluated in only 18.0% of patients who underwent a 7-year protocol biopsy. A study of the long-term protocol biopsy including DSA data is needed in the future. Fourth, our study population mostly received living-kidney transplantation and included a low number of sensitized recipients, while patients who had an episode biopsy and those who did not receive a 7-year protocol biopsy were excluded. The patients with a second or more kidney transplantation were included in the population that did not undergo a 7-year biopsy. Thus, our findings have limited generalizability to other populations more susceptible to alloimmune injury and with a more prolonged cold ischemic time, but provide evidence for the usefulness of a long-term protocol biopsy in patients with a low risk of alloimmune injury.

## Conclusion

The present study determined the clinical significance of evaluating the Banff score and clinical findings at the 7-year protocol biopsy to predict the allograft outcome. At the 7-year protocol biopsy, the presence of a Banff mm lesion alone and an mm lesion with proteinuria each predicted a decline in the eGFR. The presence of a Banff mm lesion with proteinuria had a stronger negative impact on graft survival than mm without proteinuria. These results highlight the clinical significance of the 7-year protocol biopsy to predict the renal prognosis.

## Supplementary Material

Supplemental Material

Supplemental Material

Supplemental Material

Supplemental Material

Supplemental Material
