# Supplementary material for: Prognostic value of the 7-year protocol biopsy of adult kidney allografts: impact of mesangiosclerosis and proteinuria
Source: Ren Fail. 2023 Apr 11;45(1):2197499. doi: 10.1080/0886022X.2023.2197499 (PMC10116912; doi:10.1080/0886022X.2023.2197499)
Supplement: Supplemental Material [file IRNF_A_2197499_SM0638.pdf]

Supplementary text. Graft survival results.

Fig S1 (a) shows the death-censored graft survival curves comparing cg0 vs cg≥1; the graft survival was significantly worse for cg≥1 than cg0 (P=0.003). Fig S1 (b) shows the comparison of death-censored graft survival between graft sclerosis rate<median and ≥median; the graft survival did not significantly differ between the two groups (P=0.091). Fig S2 shows the death-censored graft survival curves comparing t0 vs t1 (Fig 2a), g0 vs g≥1 (Fig 2b), ptc0 vs ptc≥1 (Fig 2c), and ti<2 vs ti≥2; the graft survival was significantly worse for t≥1 than t0 (P<0.001), and for g≥1 than g0 (P=0.005); there were no significant differences in graft survival between ptc0 and ptc≥1 (P=0.337) and ti<2 and ti≥2 (P=0.089). Fig S3 shows the death-censored graft survival curves of ct<2 vs ct≥2, ci<2 vs ci≥2, ah<2 vs ah≥2, and aah<2 vs aah≥2; the graft survival was significantly worse for ct≥2 than ct>2 (P<0.001), for ci≥2 than ci<2 (P<0.001), and for ah≥2 than ah<2 (P=0.020). There was no significant difference in graft survival between aah≥2 and aah<2 (P=0.073).

Information Classification: General
