# Supplementary figures and images for: Prognostic value of the 7-year protocol biopsy of adult kidney allografts: impact of mesangiosclerosis and proteinuria
Source: Ren Fail. 2023 Apr 11;45(1):2197499. doi: 10.1080/0886022X.2023.2197499 (PMC10116912; doi:10.1080/0886022X.2023.2197499)

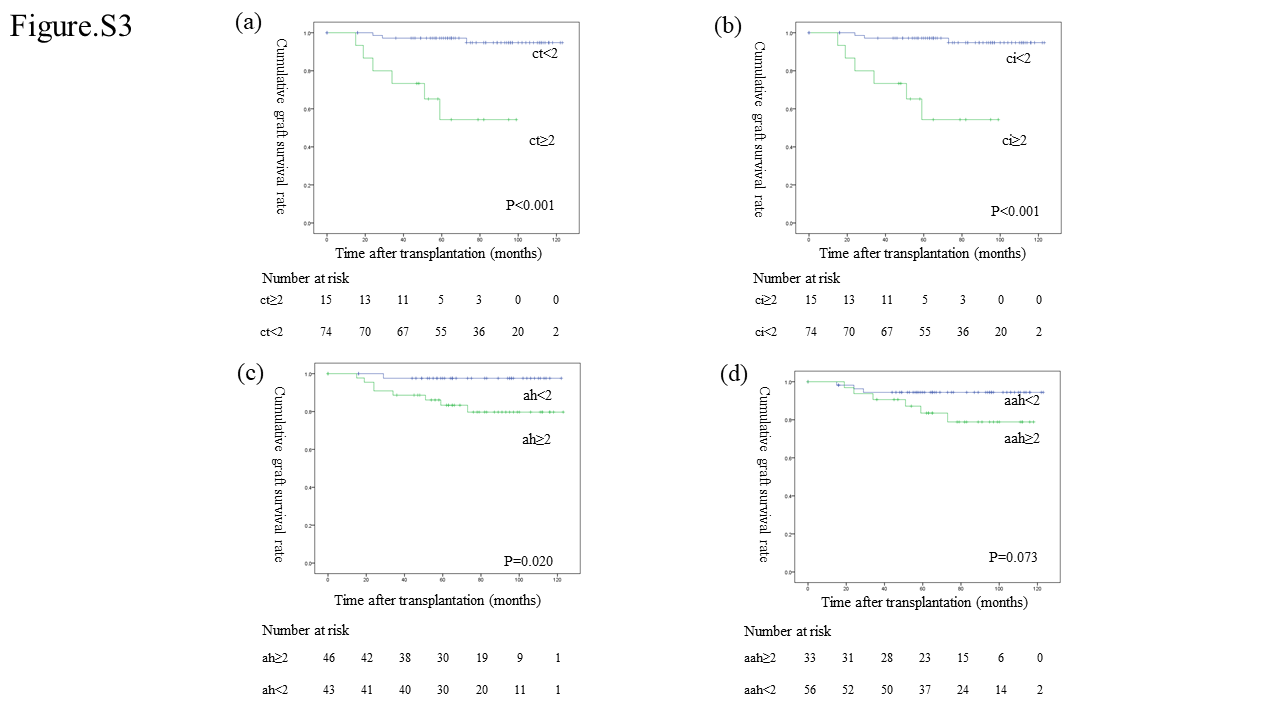

Supplement: Supplemental Material [file IRNF_A_2197499_SM0637.tif]

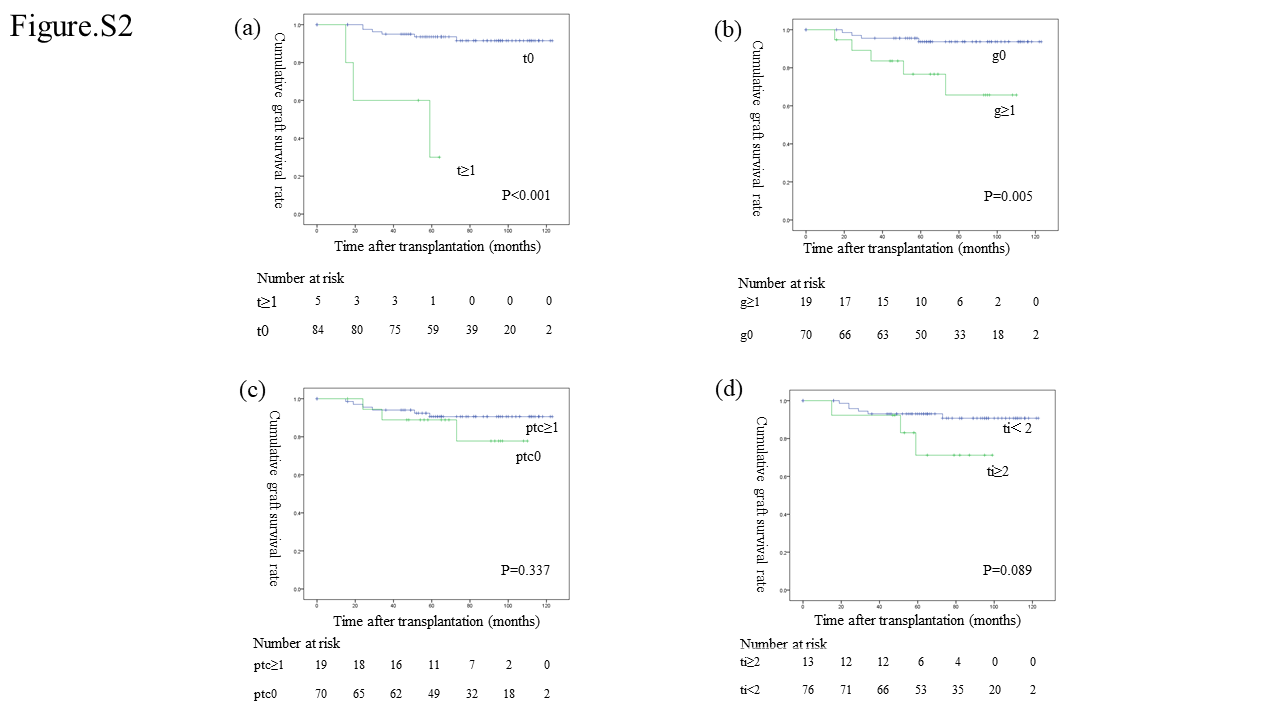

Supplement: Supplemental Material [file IRNF_A_2197499_SM0636.tif]

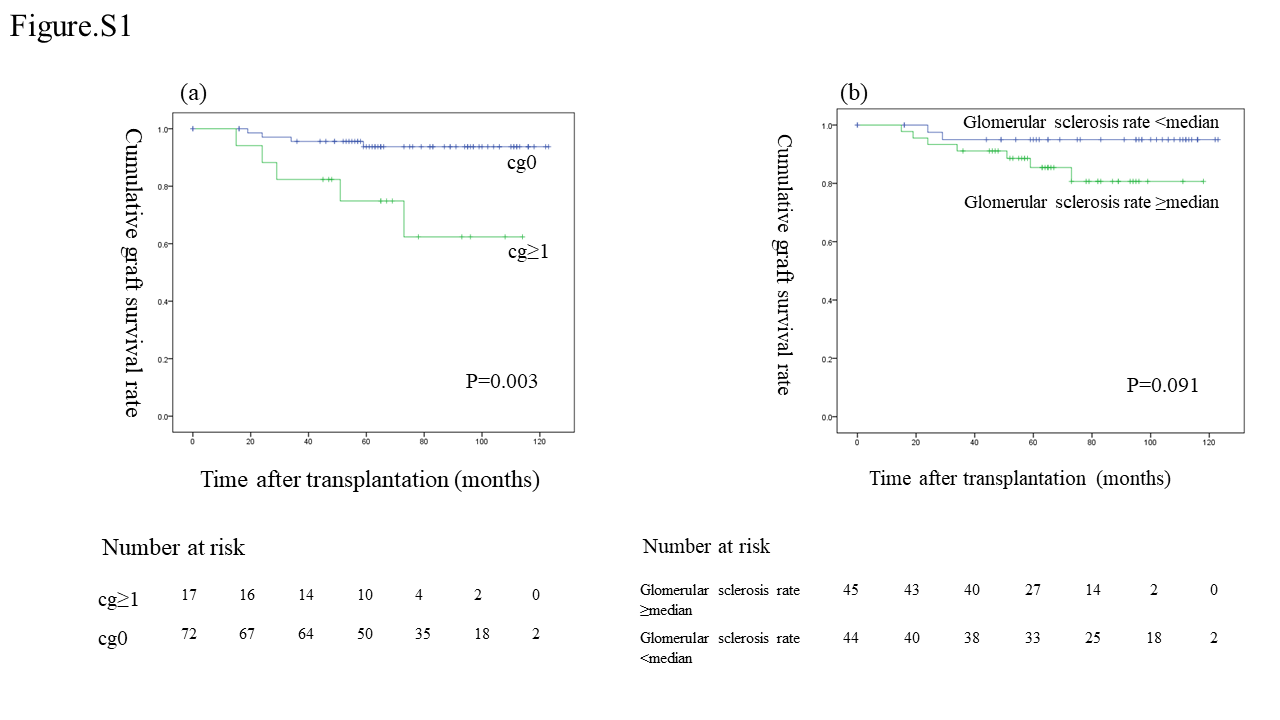

Supplement: Supplemental Material [file IRNF_A_2197499_SM0635.tif]
