# Supplementary material for: Prognostic value of the 7-year protocol biopsy of adult kidney allografts: impact of mesangiosclerosis and proteinuria
Source: Ren Fail. 2023 Apr 11;45(1):2197499. doi: 10.1080/0886022X.2023.2197499 (PMC10116912; doi:10.1080/0886022X.2023.2197499)
Supplement: Supplemental Material [file IRNF_A_2197499_SM0623.pdf]

Supplemental Table 1. Background of patients between included patients and excluded patients who did not receive a 7-year protocol biopsy

	Included patients (n=89)	Excluded patients who did not receive a 7-year protocol biopsy (n=66)	P value
Recipient age, years	40 (31, 53)	39.5 (34.8, 52.3)	0.582
Recipient sex, male/female	49/40	36/30	0.950
Donor age, years	57 (52, 64.5)	56 (44, 62)	0.067
Donor sex, male/female	42/47	24/42	0.178
Cadaveric donor, n (%)	4 (4.5)	4 (6.1)	0.724
HLA mismatches	3 (2, 3)	2 (1.8, 4)	0.991
Second or more transplantation, n (%)	6 (6.7)	13 (19.7)	0.015
ABO-incompatible	17 (19.1)	15 (22.7)	0.581
Cold ischemic time	55 (44, 75)	58.5 (45.8, 78)	0.485
Original kidney disease			
IgA nephropathy	15 (16.9)	7 (10.6)	0.270
Diabetic nephropathy	5 (5.6)	13 (19.7)	0.007
Others	46 (51.7)	33 (50.0)	0.836
unknown	23 (25.8)	13 (19.7)	0.370

Data are described as median (IQR) or n (%).

Information Classification: General
